# Stance Analysis of Distance Education in the Kingdom of Saudi Arabia during the COVID-19 Pandemic Using Arabic Twitter Data

**DOI:** 10.3390/s22031006

**Published:** 2022-01-27

**Authors:** Tahani Alqurashi

**Affiliations:** Common First Year Deanship, Computer Science Department, Umm Al-Qura University, Makkah 24382, Saudi Arabia; tmqurashi@uqu.edu.sa

**Keywords:** Arabic tweets, COVID-19, distance learning, opinion mining, stance detection

## Abstract

The coronavirus has caused significant disruption to people’s everyday lives, altering how people live, work, and study. The Kingdom of Saudi Arabia (KSA) reacted very quickly to suppress the spread of the virus even before the first case of COVID-19 was confirmed in the country. In the education sector, all face-to-face activities at public and private schools and universities were suspended, as they switched from traditional to distance learning for the entire 2020 academic year. This study collected 1,846,285 tweets to analyze the public’s dynamic opinions towards distance education in the KSA during the 2020 academic year. Several classical machine-learning models and deep-learning models, including ensemble random forest (RF), support vector machine (SVM), adaptive boosting (AdaBoost), multinomial naïve Bayes (MNB), convolutional neural network (CNN), and long short-term memory (LSTM), were tested on this data, and the best-performing models were selected to analyze the public stance towards distance education. Additionally, I correlated my analysis with the major events that were announced by the Ministry of Education (MOE). I observed that people in the KSA took some time to react and express their stances at the start of the academic year. Regarding the news, I observed that any exam-related topic attracted high engagement. In-favor stances increased when news headlines covered the topic of exams compared to other topics. The results show that the primary Saudi public stance favored distance education during the 2020 academic year.

## 1. Introduction

The breaking news of a novel coronavirus (SARS-CoV-2 or COVID-19) at the end of 2019 swiftly turned into news of a worldwide pandemic in 2020.Ever since the outbreak, people have faced unprecedented changes to their daily lives to mitigate and contain the pandemic. The first case of COVID-19 in the Kingdom of Saudi Arabia (KSA) was confirmed in March 2020, and, like many other countries, Saudi Arabia had to respond to this new challenge. Restrictions and regulations were imposed, starting with a complete lockdown, followed by curfews and the closure of schools and universities in all cities. The authorities also implemented social distancing strategies to contain and suppress the spread of the virus throughout the country. Subsequently, the public and private sectors in Saudi Arabia had to adapt to the new reality on short notice by transforming their services to an online mode by employing the newest information and communication technologies (ICTs) to meet the community’s needs and demands.

In the education sector, since the announcement made by the Minister of Education to suspend schools and universities on 9 March 2020 [1], all public schools and universities had to switch from traditional to distance learning. In higher education, institution-based learning management systems (LMSs; e.g., Blackboard) were used to manage and deliver educational activities and training content. In addition, common educational and online commercial platforms, such as Webex, Zoom, and Google Classroom, were also utilized. In contrast, public schools around the Kingdom used distance learning platforms established by the Ministry of Education (MOE): iEN [2] and Madrasati [3].

iEN is a Saudi educational satellite TV channel and a YouTube portal that comprises twelve free educational channels that broadcast daily lessons for school children from Kindergarten to Grade 12 based on the national curriculum. Madrasati is a unified learning platform for students in general education that provides many educational services and interactive tools to deliver online virtual classes, assessments, discussion spaces, and many other educational activities. It also allows for mobile and smartphone access. The use of distance learning in Saudi Arabia started in 1990, long before the COVID-19 outbreak [4]. In 2002, the first national Saudi e-learning platform was launched and contained many tailored electronic lessons and content.

However, after the emergence of the COVID-19 global pandemic, people worldwide often resorted to social media networks as their primary communication medium. A study showed that there was an increase of approximately 61% in the usage of social media platforms after the COVID-19 outbreak [5]. Social media networks have clearly become one of the most widely used means of communication for sharing thoughts, emotions, reviews, and feedback.

According to [6], approximately 79% of Saudi Arabia’s population has active social media accounts. In addition, a recent study reported that Saudi Arabia has 12.7 million active users on Twitter, which is the highest number among all Arab countries [7]. Therefore, as Twitter continues to attract more active users, more extensive efforts must be made to investigate and analyze people’s opinions and thoughts regarding specific ideas and matters of importance.

Given the unprecedented circumstances that the world has been going through, distance learning has become inevitable. Therefore, it is important to study how people respond and react to it. In light of the importance of this pressing issue, this study aims to investigate Saudi’s public opinion regarding distance education during the COVID-19 pandemic.

The objectives of this work were the following:

1. to collect and annotate Arabic tweets regarding distance learning in the KSA;
2. to train and test several classical and deep-learning models in the detection and classification of stances;
3. to evaluate and elicit the best performing model; and
4. to use the best performance model to classify the tweets in order to analyze the trends of the public stance towards distance learning in Saudi Arabia during the 2020/2021 academic year in connection with six major events.

The remainder of this paper is structured as follows: Section 2 outlines related work on Arabic stance detection. Section 3 describes the methodology. Section 4 represents my experiments and results using classical and deep-learning algorithms. Section 5 includes a discussion of the results. Section 6 illustrates the process of applying the best performance model to analyze the Saudi public stance towards distance learning during the COVID-19 pandemic. Finally, Section 7 presents concluding remarks and suggestions for future research in the area of Arabic text stance detection.

## 2. Related Work

A stance can be defined as the expression of an individual’s standpoint towards a proposition or a target [8]. Stance detection is the process of automatically extracting and determining an individual’s viewpoint towards a given controversial topic or event. An individual’s stance is mainly expressed as a written comment or text posted on an online forum, blog, or social media network (e.g., YouTube, Instagram, Facebook, and Twitter). Stance detection is a relatively new topic in the fields of opinion mining, natural language processing, and machine learning. Stance detection has been applied to investigate similar topics that have used sentiment polarity detection as a method [9,10]. Sentiment polarity detection is the process of determining and classifying an individual’s attitude on a particular topic, i.e., positive, negative, or neutral.

Wang et al. [11] state that the main difference between sentiment polarity detection and stance detection is that the former method is concerned with the popularity of the topic in light of whether it is regarded positively, negatively, or neutrally. In contrast, stance detection is concerned with the individual’s position (i.e., for or against), whether this is explicitly mentioned in the text or not. Therefore, stance detection steps up the analysis, in that it can detect if a text includes a negative sentiment but expresses an in-favor stance towards a specific topic and vice versa.

However, most efforts in Arabic opinion mining are more focused on sentiment analysis than stance detection, while the latter is, more or less, considered as a polarity sentiment analysis problem. According to recent reviews [12,13], three different approaches have been developed for Arabic sentiment polarity detection: the lexicon-based approach, the hybrid approach, and the corpus or machine learning approach (the most widely used). While the corpus/machine learning approach is applied in a supervised manner, the lexicon-based approach is usually applied unsupervised, where the data are unlabeled, and the polarity is predicted using a pre-constructed lexicon.

Al-Ayyoub et al. [14] extended the lexicon reported in [15] by including 120,000 additional terms and using a dataset consisting of 300 tweets to evaluate this extended lexicon. They managed to achieve an accuracy equal to 87%.

Oueslati et al. [12] reported that there are two directions to a lexicon-based approach. The first is to translate the available Arabic lexicon to English in order to scale down the Arabic language’s high complexity and avoid the effort of manually building a new Arabic lexicon. The second direction is to use a lexicon along with the application of machine-learning algorithms to form a hybrid approach. Recently, Aldayel et al. [16] extracted a lexicon of 1500 words translated from SentiWordNet [17] and used it to train a SVM model; they then tested how well their model classified texts into three classes (i.e., positive, negative, or neutral). This hybrid approach achieved an accuracy equal to 84%. Another recent study by Al-Twiresh et al. [18] proposed a hybrid sentiment analysis method for the Saudi Dialect dataset collected from Twitter. They used a hybrid approach that coupled the AraSenTi lexicon [19] with a SVM algorithm using a set of features evaluated using the feature-backward selection method. Moreover, they tested three different classification models: two-way, three-way, and four-way classifiers. The best performance classifier was the one constructed from the AraSenTi lexicon.

However, most Arabic sentiment polarity and stance detection research has followed a corpus/machine learning approach. This is a supervised learning approach, in which the data must be pre-labeled and processed into a suitable format for the classification algorithm. Under this approach, several classical and deep-learning algorithms have been developed.

A study by AL-Rubaiee et al. [20] applied SVM and naïve Bayes (NB) to classify 1121 Arabic tweets to investigate users’ sentiments about e-learning. They ran a two-way and a three-way classification model. In the two-way model, they classified their data into positive and negative. In the three-way model, they added the neutral class. They achieved an accuracy of 84.84% and 73.15%, respectively, using a SVM algorithm.

A more recent study by Aljarah et al. [21] used a sentiment machine-learning approach to investigate hate speech in 1633 Arabic tweets. They ran SVM, NB, decision tree (DT), and Random Forest (RF) models. First, the text was labeled into two classes: positive if it contained hate speech and negative otherwise. Next, they combined several word-embedding techniques: Bag-of-Words (BoW), Term Frequency (TF), and Term Frequency-Inverse Document Frequency (TF–IDF). They achieved an accuracy of 91% using the RN model.

A more recent study by Alhajji et al. [22] focused on analyzing Saudi Tweets. The study aimed at analyzing Saudi’s public opinions regarding COVID-19 preventive measures. Approximately 53,000 tweets were collected, and a unigram NB model was run to classify sentiments into two classes (i.e., positive and negative). They achieved an accuracy level equal to 89%. Many researchers have attempted to apply deep-learning models to solve and analyze Arabic sentiment problems, including [23,24]. In [23], they conducted several different experiments to test different deep-learning algorithms using the BoW text representation and some lexical features. Algorithms, such as recursive auto-encoder (RAE), deep belief networks (DBN), and deep auto-encoder (DAE), were used. A study by Alayba et al. [24] compared CNN to classical machine learning approaches, in particular, SVM and NB. Their results showed that the SVM outperformed the other models, achieving an accuracy equal to 91%.

Many studies used deep-learning models with AraVec pre-trained word embedding, including [25,26,27]. In [26], they compared the performance of fmy different deep-learning algorithms using AraVec as the embedded input layer; the algorithms were CNN, Bidirectional LSTM (Bi-LSTM), Bi-LSTM with an attention mechanism, and a combined CNN-LSTM architecture. They found that in detecting hate speech, coupling CNN with AraVec achieved an F1 score of 84%. Similarly, in [27], they used AraVec as the embedded input layer and used a combined CNN-LSTM architecture model. As a result, they achieved an F1 score of 64%, which they claimed outperformed state-of-the-art algorithms for the Arabic Sentiment Tweets Dataset (ASTD) [28].

Recently, ref. [25] compared the use of AraVec and TF–IDF as feature representations using 15 different classical and deep-learning models (including CNN, LSTM, BLSTM, gated recurrent unit [GRU], RF, and SVM). They concluded that using TF–IDF performed better than other word-embedding classical algorithms.

A more recent study by Aljabri et al. [29] applied sentiment analysis using machine-learning techniques to investigate people’s attitudes about the topic of distance learning in Saudi Arabia using Twitter data. They collected approximately 14,000 tweets to use as a sample in building a number of classical machine-learning algorithms, including SVM, NB, k-nearest neighbor (KNN), logistic regression (LR), and XGBoost (XGB). They achieved an accuracy of 89.9% using LR with unigram term frequency-inverse document frequency (TF–IDF) as a feature extraction method. Furthermore, they used this method to predict the sentiment of unlabeled tweets and analyzed them at two education levels (general school and higher education). They concluded that there was an overall positive opinion regarding distance learning at the general school level, whereas there was a negative opinion at the higher education level.

Table 1 summarizes of the most recent research about corpus/machine learning approaches in terms of dataset size, classification technique, and feature extraction technique used, as well as the accuracy of the best-performing algorithm.

## 3. Methodology

**Figure 1:**
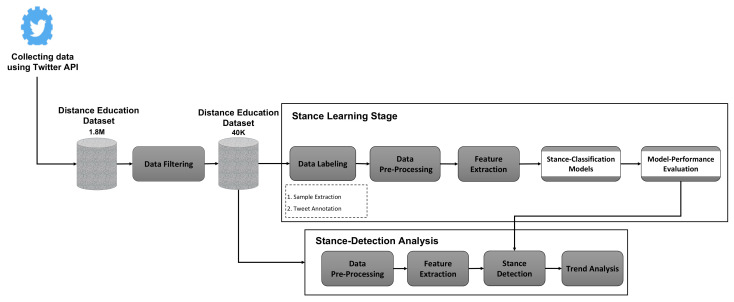
The adaptive Stance Analysis Approach including three main stages.

In this study, I implemented a three-stage methodology to analyze the Saudi public opinion regarding distance education during COVID 19 shown in Figure 1, which was adapted from [30]. In the first stage, data collection, the Twitter application programming interface (API) was used to collect relevant data. The second stage, stance learning, consisted of five steps: data labeling, data pre-processing, feature extraction, stance classification models, and model-performance evaluation. The final stage, stance detection analysis, is where I analyzed the trends and patterns related to the Saudi public opinion regarding distance education. The following subsections explain the methodology stages in more detail.

### 3.1. Data Collection Stage

I used the Twitter streaming (API) to collect Arabic tweets. I began by scraping relevant hashtags regarding distance education in the region of Saudi Arabia during the considered period. The hashtags scraped included: التعليم ـ عن ـ بعد، #الدراسةـ عن ـ بعد، #منصة ـ مدرستي #

Moreover, I noticed several other closely related hashtags that did not explicitly mention distance learning; they were as follows: #نعودـ بحذر ، #التباعدـ الاجتماعي ، #الدراسة ـ مستمرة ، #كورونا ، # جائحة، # الحظرـ الكلي #كوفيد ـ٩١ #تفشي، #الاختبارات #الفصل ـ الدراسي ـ الاول ، #الفصل ـ الدراسي ـ الثاني

Table 2 shows the list of the keywords used to collect tweets related to distance learning in Saudi Arabia.

The tweets collected covered the period starting 15 August 2020, the date of the announcement made by the MOE suspending all schools and universities in the KSA and the switch to distance learning, up until the end of March 2021, the last month in the 2020/2021 academic year in Saudi Arabia.

As shown in Table 3, the total number of collected tweets was 1,846,285. After filtering spam text, advertisements, non-Arabic tweets, duplicate tweets, and retweets, the remaining number of tweets in the cleaned dataset was 464,410.

### 3.2. Stance Learning Stage

The main aim of this stage was to build several stance classification models that could learn from human-annotated data to be used in testing the collected data in the next stage.

There are five steps in this stage as shown in Figure 1, which are: Data Labeling, Data Pre-Processing, Feature Extraction, Stance-Classification Models, and Model-Performance Evaluation.

#### 3.2.1. First Step: Data Labeling

In this step, two tasks were performed: Sample Extraction and Tweet Annotations. In the first task, a sample of 4348 tweets were extracted for learning purposes. This sample had a total of 17,232 unique words. I asked three native Arabic speakers to annotate the sample data in the second task.

For the annotation task, two annotators were first asked to label each tweet, either In-favor or Against distance learning in Saudi Arabia. If the two annotators disagreed regarding the classification of a particular tweet, a third annotator was also asked to label it. Table 4 shows the statistics for the manually annotated dataset. As displayed, there was a total of 4348 tweets. My annotated dataset is unbalanced: the In-favor class has 1751 more tweets than the Against class. Table 5 shows a sample of In-favor and Against tweets along with their English translations.

#### 3.2.2. Second Step: Data Pre-Processing

As each tweet is limited to 280 characters, users usually tend to write in an informal way, including special characters, numbers, links, and emojis. Therefore, Twitter raw data is very noisy and must be pre-processed before implementing any analysis. Moreover, the Arabic language is vibrant and rich, and users tend to use informal Arabic, not standard Arabic.

Therefore, several measures were implemented in the Data Pre-processing step as follows:

- Unwanted characters were removed from the tweets, such as links, emojis, special characters (#, %, &), Arabic diacritics, punctuation marks, and numbers.
- Tweets written in a language other than Arabic were deleted.
- Text correction was performed using the TextBlob library in Python [31].
- I normalized the Arabic text as follows:
  - إ, أ and آ was replaced with ئ; ا was replaced with ى; ا was replaced with ة; ي was replaced with ؤ; ه was replaced with و; and كـ was replaced with ك.
- Duplicate characters were removed, as in جمييييييل, مرررره, and راااااائع.
- Arabic stop words were removed, such as على, في, من, and الى.

#### 3.2.3. Third Step: Feature Extraction

In the Feature Extraction step, the aim was to extract the features to transform the raw tweets into a structured form suitable for the machine-learning algorithms. I constructed a space vector based on Term Frequency–Inverse Document Frequency (TF–IDF) and performed word embedding. The following sections explain the tasks implemented.

##### Space Vector Based on TF–IDF

In this task, TF–IDF was constructed by combining two scores: the term frequency (TF) and the inverse document frequency (IDF). This method reduces the weight of words that are repeated frequently and increases the weight of words that are repeated very rarely. TF measures the frequency of a word in a tweet. IDF measures whether a word is common or rare. The TF–IDF is calculated as shown in the following:

(1) TF–IDF(w,t,D)=TF(w,t)×IDF(w,D),

where TF(w, t) calculates the number of times a word w appears in a tweet t.

IDF is defined as follows:

(2) $$\mathrm{IDF}(w,D)=\log\frac{\left|D\right|}{\mathrm{df}(w)},$$

where D is the total number of tweets in the dataset and df(w) is the number of tweets in which word w appears in D.

##### Word Embedding

For the word embedding task, I used two AraVec models, which were previously built using a multidimensional vector space, namely the Continuous Bag-of-Words (CBoW) technique and the skip-gram technique. The models were trained using a large Arabic Twitter dataset of approximately 77,600,000 Arabic tweets [32]. Each tweet was represented as a 2D vector with a dimension of *m* × *d*, where m is the number of words in the tweet and d is the length. As I used the AraVec CBoW and skim-gram models with a dimension of 300, I set d=300. I fixed all of the tweets with the same size of 44 by padding each tweet’s representation with zeros, following the same approach as in [33]. Therefore, each tweet was of the size 44 × 300.

Furthermore, I tested using a weighted AraVec model combined with the TF–IDF scores for each word in a tweet by multiplying the word embedding with its TF–IDF score. This procedure was performed for all the words in all the tweets. The total was then aggregated and divided by the accumulated TF–IDF scores of the words in the tweets.

#### 3.2.4. Fourth Step: Stance-Classification Models

In the fourth step, which is the stance classification models, the performance of several classification algorithms was investigated. The algorithms used were: random forest (RF), support vector machine (SVM), Adaptive boosting (AdaBoost), multinomial naïve Bayes (MNB), convolutional neural network (CNN), and long short-term memory (LSTM).

The RF technique is a popular and powerful ensemble machine-learning algorithm that uses decision trees to construct a number of individual models (forests) that can be combined using a bagging method to form the final class labels of the data [34]. To train RF, two parameters must be specified; these indicate the number of trees in the forest and the number of selected features for each node, in consideration of when each is split. One of the major advantages of random forest is that it is less prone to an over-fitting situation, even if more trees are appended to the forest [34].

The SVM method is based on a statistical learning theory used to determine the location of the decision boundaries of the most optimal separations of classes. SVM was initially designed for binary classification problems, where the two classes are linearly separable at the greatest margin between them. This margin is calculated as the sum of all the distances between the closest points of the two classes and the hyperplane. These closest points are called “support vectors” and are always small in number [35].

When the two classes are not linearly separable, the hyperplane that maximizes the margin and minimizes the number of misclassification errors selected. This trade-off is controlled by the positive user-defined parameter *c*, and it projects the data into a high-dimensional feature space using nonlinear mapping and a kernel function to reduce the computational cost. One commonly used kernel function is the radial basis function (RBF), also called the Gaussian kernel. Suppose I have a data point x and a support vector y; the linear function is calculated as the dot product of x and y. The RBF requires a parameter called γ and calculates the Euclidean distance between x and y. The RBF is calculated as follows:

(3) $$K(x,y)=\exp(-{\gamma ||x-y||}^{2}).$$

AdaBoost is an ensemble machine-learning algorithm that combines a number of weak classifiers into a final classification result formed by a weighted sum of the boosted classifier. The main objective of AdaBoost is to adaptively change the training data weights according to the results produced by the wear classifiers in each round. In fact, this adaption process forces the weak classifiers to focus on the incorrectly classified data in order to boost the performance of the final classification results [36].

The naïve Bayes method is a probabilistic classification algorithm that applies the Bayes theorem. In naïve Bayes, the classification task is performed under a strong assumption that each feature is independent from the other features. The MNB approach is used with multinomially distributed data, and it is very popular for natural language processing tasks, such as text classification problems [37].

The CNN is a type of deep neural network that has been successfully applied in many natural language processing (NLP) domains, such as text classification. In my investigation, I adopted an architecture approach similar to the one presented in [33] to build the CNN model. It consisted of a word-embedding layer constructed from the AraVec word vectors with a dimension equal to 300. The word-embedding layer was then followed by five 1D convolutional layers of various sizes (2–6) using rectified linear unit (ReLU) activation, and each of the five layers was followed by a max-pooling layer.

The output of these five layers was merged in a concatenation layer (none, 1000). A dense layer of size 128 was then passed through a sigmoid layer to produce the final classification probability results. To prevent over-fitting, I added two dropout layers, one after the max-pooling layer and another after the fully connected hidden layer, with a dropout probability of 20 as recommended in [27].

The LSTM is a Recurrent Neural Network (RNN). My experiments used a stacked bi-directional LSTMs approach, as this type of approach has proven to be more efficient than one bidirectional LSTM. One bidirectional LSTM examines only the input sequence in a forward direction, while a stacked LTSM approach combines information from both ends (forward and backward) into one single representation to learn better feature representation.

I implemented the same architecture approach used by [27], which consists of the following layers: word embedding with a dimension of 300, bidirectional LSTM, and a fully connected layer with a ReLU activation function; the final classification probability was produced using the sigmoid layer. Moreover, two dropout layers were applied: one before the fully connected layer and one after it with a drop rate of 0.2 as recommended in [27]. The LSTM hidden state dimension was set to 200. The number of hidden units was set to 30. The learning rate was set to 0.001. The number of epochs was set to five, and the batch size was set to 60.

#### 3.2.5. Fifth Step: Model-Performance Evaluation

As my annotated dataset was imbalanced, I evaluated the performance of the machine-learning algorithms based on the most popular metrics: recall, precision, F-measure, and the area under the receiver operating characteristic (ROC) curve (AUC). They are presented below.

First, it is necessary to define the following:

- A true positive (TP) is the number of real In-favor tweets classified as In-favor.
- A false positive (FP) is the number of real Against tweets classified incorrectly as In-favor.
- A false negative (FN) is the number of real In-favor tweets incorrectly classified as Against.
- A true negative (TN) is the number of Against tweets correctly classified as Against.

Recall represents the ratio of the correctly predicted positive tweets to the total number of tweets in the actual class. It is calculated as follows:

(4) $$\mathrm{Recall}=\frac{\mathrm{TP}}{\mathrm{TP}+\mathrm{FN}}.$$

Precision represents the ratio of correctly predicted positive tweets to the total number of predicted positive tweets. It is calculated as follows:

(5) $$\mathrm{Precision}=\frac{\mathrm{TP}}{\mathrm{TP}+\mathrm{FP}}.$$

F-measure represents the weighted average, and it is calculated as follows:

(6) $$F\text{-}\mathrm{measure}=2\times \frac{\mathrm{Precision}\times \mathrm{Recall}}{\mathrm{Precision}+\mathrm{Recall}}.$$

AUC measures the extent to which the classifier is capable of distinguishing between classes [38], and it is calculated as follows:

(7) $$\mathrm{AUC}=\sum_{i\in (\mathrm{TP}+\mathrm{FP}+\mathrm{FN}+\mathrm{TN})}\frac{\left({\mathrm{TPR}}_{i}+{\mathrm{TPR}}_{i-1}\right)\times \left({\mathrm{FPR}}_{i}+{\mathrm{FPR}}_{i-1}\right)}{2},$$

where the true positive rate (TPR) is the same as Recall (defined in Equation (4)). The false positive rate (FPR) is calculated as follows:

(8) $$\mathrm{FPR}=\frac{\mathrm{FP}}{\mathrm{FP}+\mathrm{TN}}.$$

It is worth mentioning that all the above metrics range between zero and one, where one represents a classifier with perfectly predicted results and zero represents a performance worse than a random classifier. Thus, the closer the value is to one, the better the model predicts the tweets as being In-favor or Against.

### 3.3. Stance Detection Analysis Stage

After selecting the best-performing model, I used it to analyze the evolution of the public stance towards distance learning in Saudi Arabia during the period of interest. My stance analysis was correlated with the major announcements made by the Saudi MOE regarding the suspension of schools and universities across the Kingdom due to coronavirus concerns.

## 4. Experiments and Results

I ran all my experiments using the high-level technical computing language Python 3 version 3.7.6 on Apple Macintosh computer 2 GHz Quad-Core Intel Core i5 with 16 GB memory. I used the scikit-learn Python library [39] to implement the classical machine-learning classification algorithms. For the deep-learning algorithms, I used the Keras library with TensorFlow as the back-end [40].

I ran my experiments using 5-fold cross-validation, a widely used approach to compare classifiers in machine learning. First, the classification model was trained using fmy folds as the training data, while the constructed model was validated using the remaining fold of the annotated dataset. This was performed five times with each fold serving as the validation set once. The average values were then computed after all the runs were completed. Table 6 shows the results of the considered algorithms, and the following subsections discuss these results further.

### 4.1. The Results of Classical Machine-Learning Algorithms

To achieve my aim, I developed an Arabic natural language processing pipeline to determine the best parameters using a grid-search approach. The developed pipeline includes the following tested parameters:

- Different number of features, including all generated features, 1500, 2000, 3000 and 5000.
- Different n-gram combinations for the string vectorizer including unigrams (1-1), unigrams + bigrams (1-2), unigrams + bigrams + trigrams (1-3), bigrams (2-2), bigrams + trigrams (2-3), and trigrams (3-3).
- Different values for the document frequency threshold (maxDF), including 0.5, 0.75, and 1.0.

I experimented with different settings for the classification algorithms with a different number of estimators (n-estimators). I tested 16 and 32 estimators. For the kernel function of the SVM, I tested the linear and the radial basis function (RBF) kernel. For the SVC algorithm, the values one and 10 for the regularization parameter were also tested.

**Figure 2:**
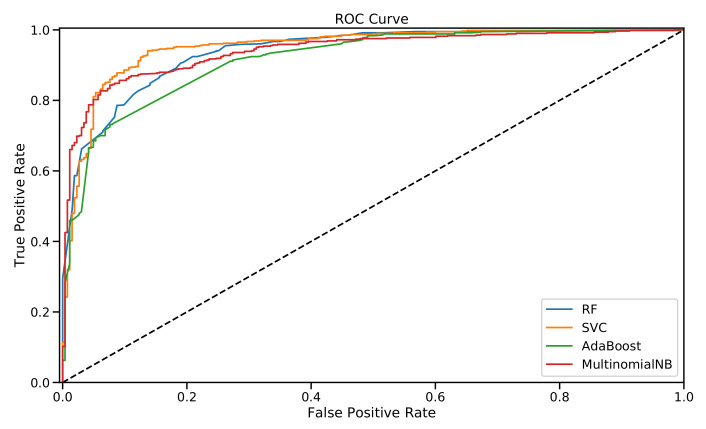
Visualization of the ROC curves of the classical machine-learning algorithms using TF–IDF features.

Table 6 displays the best performance achieved using the parameters determined using the grid search. I also generated the ROC curves shown in Figure 2 for the best performance achieved by the classical machine learning classifiers using the TF–IDF features.

The Random Forest classifier (RF) achieved the best results when the maximum number of features was reduced to 5000, including only unigrams as the features, keeping maxDF equal to 1.0, and using 32 estimators.

In the case of the AdaBoost classifier, the best performance was achieved when the maximum number of features was reduced to 5000, configured with the n-gram model (1,3), keeping the maxDF threshold equal to 0.75 and n-estimators equal to 32. The performance was equal to 0.817 and 0.916 for the F-measure and AUC score, respectively. This had the worst performance compared to the other three classifiers using TF–IDF as the feature construction method.

In the case of the MNB classifier, the best results were achieved when the maximum number of features was reduced to 5000, while including only unigrams as features and keeping maxDF equal to 0.5. This result produced values equal to 0.818 and 0.940 for the F-measure and AUC score, respectively. This had the second best performance compared to the other three classical machine classifiers in terms of AUC score.

In summary, as displayed in Table 6, the best-achieved results were reported for the SVC algorithm with 0.859 and 0.951 being measured for the F-measure and AUC score, respectively. The results of the SVC algorithm were achieved using the RBF kernel function and a maxDF threshold equal to 0.75. Moreover, the maximum number of features was reduced to 3000 and included only unigrams as the features.

The experiments highlighted that the best results for three out of the fmy classifiers were achieved using unigrams when constructing TF–IDF features while reducing the maximum features to 5000.

Consequently, I tested the SVC classifier with word embedding. I used AraVec CBoW and skip-gram models as the feature construction method [32]. Surprisingly, using the AraVec CBoW and skip-gram models with the SVC classifiers did not improve the results. Instead, it performed poorly compared to all the fmy classical machine-learning algorithms that used TF–IDF as the feature construction method.

I observed that using the weighted AraVec CBoW and skip-gram models improved the results dramatically with the AUC scores increasing to 0.406 and 0.408, respectively. Although there was an observed improvement when using the weighted AraVec CBoW and skip-gram models, the SVC classifier using TF–IDF still outperformed these models.

### 4.2. The Results of Deep Learning

The adaptive moment estimation (Adam) approach was applied to the learning models’ parameters with varying learning rates; a batch size of 60 and 500 epochs were used to optimize the cross-entropy loss.

As can be seen from Table 6, the CNN model that achieved the best result was the one that used the AraVec skip-gram model as a word vector, which reached values of 0.834 and 0.938 for the F-measure and AUC score, respectively. Furthermore, I found similar results for the LSTM model in which the performance using the same word vector model increased, reaching 0.836 and 0.944 for the F-measure and the AUC score, respectively.

The deep learning results were not better than the SVC model using TF–IDF as the feature construction method. However, both of the deep-learning models (CNN and LSTM) that used the word embedding created through the AraVec approach performed better than the classical SVC classifiers that have used the same word-embedding approach.

## 5. Discussion

All in all, using TF–IDF with SVM performed better than using the AraVec models. This result corroborates the results of the study on Arabic text by [25]. Moreover, the results highlight the fact that SVM outperformed other classical learning algorithms, including AdaBoost, RF and Multinominal NB. This also corroborated the results found in [20,24]. In terms of deep learning, I found that the weighted AraVec model can dramatically improve the performance of the SVM, whereas it had only a slight improvement when used as an input layer for the deep-learning algorithm. In conclusion, my study demonstrated that SVM using TF–IDF as feature representation outperformed other machine-learning algorithms.

## 6. Analyzing Social Media

My experiments showed that the best performance model among all those tested was the SVM model trained using all the annotated tweets. Therefore, I used that same model to analyze the trends of the public stance towards distance learning in Saudi Arabia during the 2020/2021 academic year. In the following subsection, first, I state the major events that had taken place. I then discuss my collected data concerning the news made to announce the major events.

### 6.1. Major Events

First, I searched (using Google) for the major announcements made by the Saudi MOE regarding the suspension of schools and universities across the Kingdom due to coronavirus concerns during my period of interest. There were six major announcements listed below:

- A1:Announcement of the switch to remote learning for the Hijri year 1442 for seven weeks. (https://www.spa.gov.sa/viewfullstory.php?lang=ar&newsid=2120893 (accessed on 15 August 2020)).
- A2: Announcing the continuation of distance learning for the remaining weeks of the first semester. (https://www.spa.gov.sa/viewfullstory.php?lang=ar&newsid=2142888 (accessed on 8 October 2020)).
- A3: Adoption of a mechanism for performing the first semester’s final exams for general education students and administering the grades. (https://www.spa.gov.sa/2147922 (accessed on 24 October 2020)).
- A4: Announcing the continuation of distance learning for the second semester until the tenth week of the semester. (https://www.moe.gov.sa/ar/mediacenter/MOEnews/Pages/PE-2547S.aspx (accessed on 13 January 2021)).
- A5: Distance learning will continue until the end of the school year. (https://www.moe.gov.sa/ar/mediacenter/MOEnews/Pages/ERC1442-23.aspx (accessed on 22 February 2021)).
- A6: Bringing forward the exam period for the second semester to start on the first day of Ramadan for the primary school students and the sixth dayof Ramadan for middle and secondary school students. (https://www.moe.gov.sa/ar/mediacenter/MOEnews/Pages/th1442-89.aspx (accessed on 29 March 2021)).

### 6.2. Trend Analysis

The SVM model with the best performance score was applied to the cleaned tweet dataset to classify each tweet into either the In-favor or Against classes regarding their stance on distance learning. Because most of the events that the MOE had announced included start and end times of the suspension of schools and universities (i.e., they involved a switch regarding distance learning) and were bounded by a certain number of weeks, the analysis was performed on a week-by-week basis that was divided into two parts. The first part of the analysis included three weeks before the academic year (3 WB, 2 WB, and 1 WB) and the 18 weeks of the first academic semester. The second part of the analysis included the two weeks of the half-term holiday (HTW1 and HTW2) and the 11 weeks of the second academic semester.

**Figure 3:**
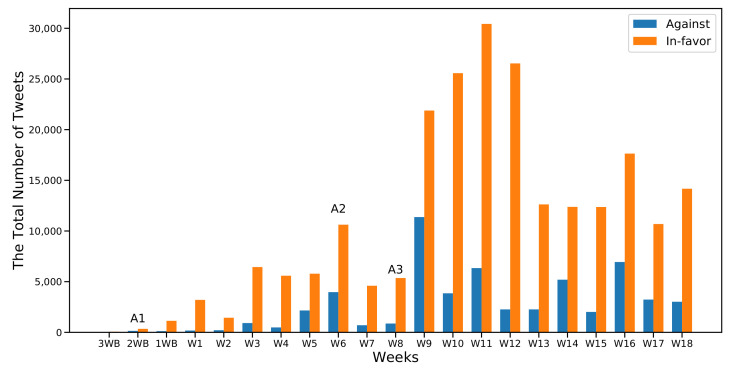
Distribution of cleaned tweets collected in the first semester.

**Figure 4:**
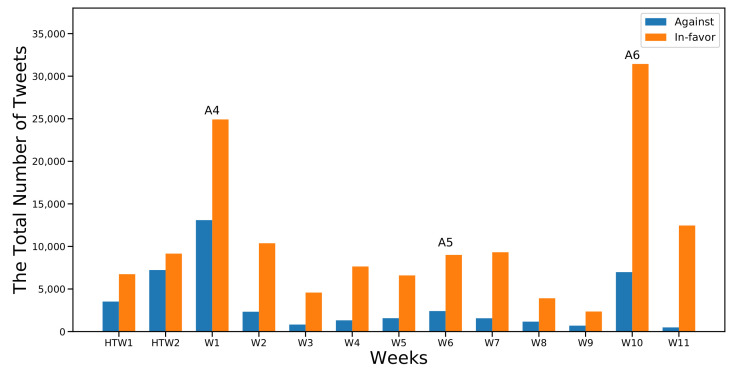
Distribution of cleaned tweets collected in the second semester.

Figure 3 and Figure 4 show the first and the second part of the analysis, respectively, along with the frequencies of the two considered classes (In-favor, Against). Moreover I marked the occurrence of the six major events that were previously identified in Section 6.1. As expected, the number of In-favor tweets exceeded the Against tweets.

As observed in Figure 3, before the start of the first semester, the collected and cleaned number of tweets was below 5000 even during the announcement of the A1 news. Most of the tweets were in favor of distance learning. However, looking at the total number of unprocessed tweets during the same period, I found the number of raw tweets was much higher than the number of clean ones. This phenomenon can be explained by the fact that many advertising companies and users take advantage of trending hashtags during certain periods to attract traffic to their accounts. Moreover, people tend to take some time to react and express their stances regarding new and unforeseen situations. In the case of my data, it appeared to take approximately two weeks from the start of the first semester.

Another explanation for the lack of user engagement at the beginning of the first academic semester was that event A1 stated a time limit of seven weeks. Therefore, at the beginning of the pandemic, it was reasonable to believe that people did not expect the distance learning to be used for long and did not, in turn, feel the urge to express any stance towards it. However, as weeks passed and distance learning became the new normal, people’s stances regarding it appeared to evolve, reaching more than 10,000 tweets, with 72% having In-favor stances by the announcement of event A2 in W6.

However, the most notable differences between the two categories were recorded at W10, W11, and W12 of the first semester after event A3 (Figure 3) and at W10 in the second semester at the same week of the announcement of event A6 (Figure 4). A3 was the announcement regarding the mechanism of administrating the first semester’s final exams. A6 was the announcement on the advancement of the second semester exams.

It can be assumed that any exam-related topic would attract more engagement and drive people to express their stances, as appears to be happening here. In-favor stances increased more when the topic was related to exams when compared to other topics; 79.9% and 85.5% for events A3 and A6, respectively.

Moving to the second part of the analysis, I found that during the two-week half-term holiday (HTW1 and HTW2) (Figure 4), the difference between In-favor and Against was not significantly higher compared with other weeks. The difference reached percentages of 34% and 44% in HTW1 and HTW2, respectively. The latter was the highest percentage recorded for the Against class in the analyzed period.

After the announcement of event A4 in W1 of the second semester, the percentage of Against stances decreased by 10% (the In-favor class reached 65% out of a total of 38,012). A4 was the event regarding the continuation of school suspension and distance learning until the tenth week of the second semester. Students, at this time, had been engaged in distance learning since the start of the academic year in September (fmy months). The total number of tweets dropped to 12,000 at W2 and gradually continued to decrease until the occurrence of event A5 at W6. Despite the decrease in the total number of tweets, there was a slight increase in the percentage of the In-favor class, which reached 80% in W5 after the announcement of event A5. It increased by 2% and 5% in W6 and W7, respectively.

Nevertheless, event A6 at W10 dramatically increased the total number of clean tweets. The count reached more than 38,000 tweets, with 81% classified as In-favor. This event, as explained earlier, was regarding an exam-related topic. Therefore, I could presume that major events strongly attract people to express their stances compared to their absence during other time intervals.

**Figure 5:**
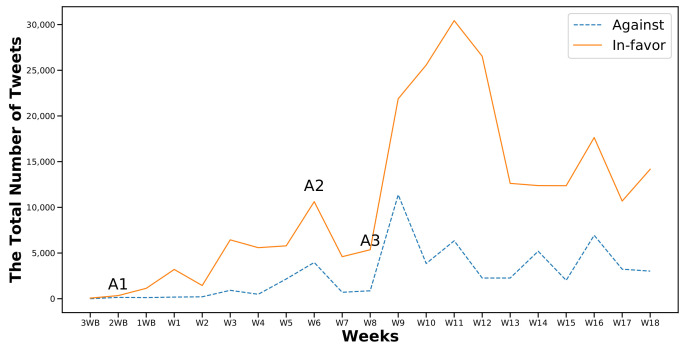
Cumulative weekly cleaned tweets for the first semester of the 2020 academic year.

**Figure 6:**
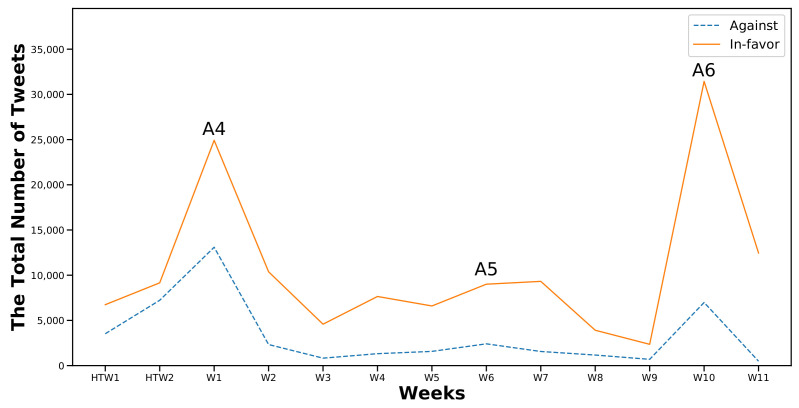
Cumulative weekly cleaned tweets for the second semester of the 2020 academic year.

Comparing the numbers of Against and In-favor tweets, I noticed that there were fluctuations during the entire period of interest as shown in Figure 5 and Figure 6. The curve of the Against class was flat except during 3WBF to W4 in the first semester and W3 to W9 in the second semester. In contrast, the curve of the In-favor tweets recorded its highest peaks at W11 in the first semester and after event A6 at W10 in the second semester.

**Figure 7:**
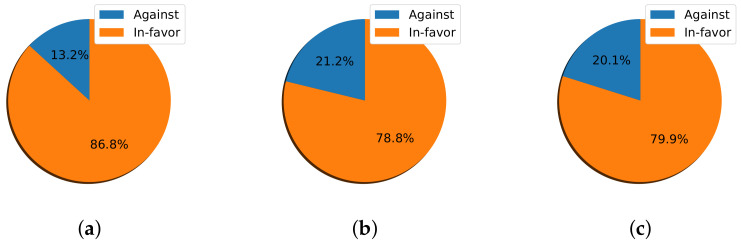
The percentage of In-favor and Against tweets of the three events occurring in the first semester of the 2020 academic year. (**a**) A1. (**b**) A2. (**c**) A3.

**Figure 8:**
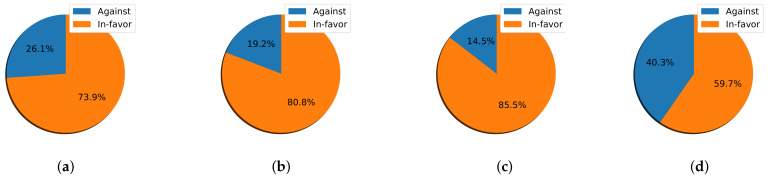
The percentage of the In-favor and Against tweets of the three events occurring in the second semester of the 2020 academic year. (**a**) A4. (**b**) A5. (**c**) A6. (**d**) HTW1&2.

I calculated the percentage of tweets expressing In-favor and Against stances for a particular event from its announcement day until the next event (Figure 7 and Figure 8). The highest percentage of In-favor stance tweets was recorded for event A1, reaching 86.8% in the first semester; this was followed by event A6, reaching 85.5% in the second semester. In contrast, the lowest percentage of In-favor stance tweets of 59.7% was recorded during the two-week half-term holiday, followed by event A4, which reached 73.9%.

Considering the half-term holiday (Figure 8d), as expected, there was an increase in the percentage of Against stance tweets (40.3% of a total of 26,650 tweets). This was the highest percentage recorded in the Against class during the whole analysis period. Therefore, I can make an assumption that the holiday allowed people to express their stances more vividly, given that they had more time to think of the situation and evaluate the new reality of distance learning.

After the announcement of A4, the In-favor stance tweets increased by 14.2% (Figure 8a). This further increased by 6.9% and then by 4.7% after the announcements of A5 and A6, respectively. A5, announced at W6 of the second semester, declared the continuance of distance learning until the end of the 2020/2021 academic year. People, by this time, had already been using distance learning for approximately six months and had presumably become used to it and more familiar with the imposed situation.

By W10 of the second semester (precisely on 24 March 2021), the Saudi Ministry of Health (MOH) announced that “more than 3.5 million COVID-19 vaccine doses had been administered through over 500 vaccination centers across the Kingdom”. The news of the vaccine came as a great relief to the public and fueled hopes of life getting back to normal. In this context, I expected an increase in the percentage of the Against class regarding distance education. However, during the same week, A6 was announced mentioning exams, and instead, I observed a significant increase in the In-favor stance tweets.

## 7. Conclusions, Limitations, and Future Research

This study analyzed Saudi public stances regarding distance education in the KSA during the COVID-19 pandemic, particularly during the 2020 academic year. I implemented an approach that consisted of three stages: data collection, stance learning, and stance detection. Accordingly, RF, SVM, AdaBoost, MNB, CNN, and LSTM were used in the stance learning stage, and their performances were evaluated and compared. The results show that the best-performing algorithm was SVM using TF–IDF as a feature construction method, which achieved 0.859 and 0.951 for the F-measure and AUC scores, respectively. Moreover, I was able to correlate my analysis with six major announcements that the MOE made regarding the suspension of schools and universities across the KSA due to coronavirus concerns during my period of interest. The results of my study could help decision makers in the MOE assess public opinions towards distance learning. Moreover, the study implemented a stance-detection methodology that was adapted particularly for the Arabic language, which can be used by any government to assess public stances toward their services and rules.

While this work has demonstrated a potential approach to analyzing and detecting public stances in Arabic language texts, there are a number of limitations that need to be acknowledged. First, in this study, I used Modern Standard Arabic (MSD) keywords to collect my dataset. However, most users write in their own local dialect on social media. Therefore, it is suggested that further research should consider expanding this paper’s data to include some common Saudi dialect keywords and accordingly develop an algorithm based on Arabic natural language processing to detect and analyze Saudi dialect tweets. Second, in this study, I only focused on Arabic text; a further study should consider including the analysis of emojis in order to improve the stance analysis results. Moreover, one limitation of this study was that I only considered two-way classification problems.

In future research endeavors, in terms of the stance-classification model, I plan to improve it further by using different word-embedding models, such as Arabic bidirectional encoder representations from transformers (AraBERT), and also by considering three-way classification problems. Moreover, I plan on using a combination of machine-learning algorithms to form an ensemble classification method in order to improve classification accuracy. I also plan on building a Saudi dialect lexicon from my collected dataset and will consider a lexicon-based approach to detect and analyze public stances. One interesting observation from my analysis was that any exam-related topic attracted more Saudi public engagement than the other topics. Therefore, in the future, I plan to investigate a wider range of related topics covered in the news headlines and expand the covered area to include different countries.

## Figures and Tables

**Table 1:** Summary of the most recent research about corpus/machine learning approaches.

Reference	Dataset Size	Classification Technique(s)	Feature Extraction Technique(s)	Accuracy
[20]	1121	SVM, NB	TF–IDF, N-grams	73%
[21]	1633	SVM, NB, DT, RF	BoW, TF, TF–IDF	91%
[22]	53,000	NB	unigram	89%
[23]	3795	RAE, DBN, DAE	BoW	73%
[24]	2026	CNN, SVM, NB	unigram, bigrams, TF–IDF	91%
[25]	8000	CNN, LSTM, Bi-LSTM, RF, SVM	AraVec, TF–IDF	73%
[26]	15,050	CNN, Bi-LSTM, CNN-LSTM	AraVec	84%
[27]	10,000	CNN-LSTM	AraVec	64%
[28]	10,000	MND, BNB, SVM	TF–IDF	69%
[29]	14,000	LR, NB, KNN, XGB, SVM, RF	N-gram, TF–IDF	89%

**Table 2:** The list of keyword used in hashtags to collect tweets in my dataset.

Keyword	English Translation
التعليم عن بعد	Distance Education
الدراسة عن بعد	Distance Studying
منصة مدرستي	My School Platform
نعوذ بحذر	Back with Caution
التباعد الاجتماعي	Social Distancing
الدراسة مستمرة	Continuous Study
كورونا	Corona
كوفيد٩١	COVID 19
جائحة	Pandemic
الفصل الدراسي الاول	First Semester
الفصل الدراسي الثاني	Second Semester
الاختبارات	Exams

**Table 3:** The list of hashtags used to collect tweets in our dataset.

Year	Month	Tweets	Cleaned
	August	110,439	2059
	September	41,785	22,028
2020	October	92,564	61,079
	November	162,739	119,078
	December	850,739	78,465
	January	99,489	78,369
2021	February	58,035	34,873
	March	430,495	68,459
Total		1,846,285	464,410

**Table 4:** Statistics for the manually annotated dataset.

Class	Total Number	Percentage
In-favor	3063	70.45%
Against	1312	30.17%
Total	4348	100.00%

**Table 5:** Example of an In-favor and Against tweets along with their English translations.

Label	Language	Tweet Example
In-favor	AR	الخيار واضح لكل عاقل التعليم عن بعد طبعا سلامة الابناء اهم ووسائل التعليم بالمنزل متاحه الحمد لله
	En	The choice is clear to every sane person, distance education, of course. The safety of the children is the most important. Thank God education at home is available.
Against	AR	إضافة إلى عدم تطوره الاجتماعي والعاطفي بشكل اعتيادي، يؤثر التعليمعنبعد على مسار الطفل الأكاديمي أيضاً، وهو ما يؤكده الخبراء كونه أمرلا يستهان به
	En	In addition to his lack of normal social and emotional development, distance education affects the child’s academic path as well, which is confirmed by experts as a matter to be reckoned with.

**Table 6:** The overall performance of the classical and deep machine-learning classifiers.

Classifier	Precision	Recall	F-Measure	AUC Score
RF				
n_estimators = 32, maxDF = 1.0,	0.884	0.837	0.855	0.939
max_features = 5000, ngram_range = (1, 1).				
SVC				
C = 1, kernel = rbf, maxDF = 0.75,	0.892	0.838	0.859	0.951
max_features = 3000, ngram_range = (1, 1).				
AdaBoost				
n_estimators = 32, maxDF = 0.75,	0.837	0.803	0.817	0.916
max_features = 5000, ngram_range = (1, 3).				
MultinomialNB,				
maxDF = 0.5, max_features = 5000,	0.863	0.795	0.818	0.940
ngram_range=(1, 1).				
SVC				
AraVec-SG	0.349	0.500	0.411	0.521
SVC				
weighted AraVec-SG	0.862	0.840	0.850	0.929
SVC				
AraVec-CBoW	0.349	0.500	0.411	0.505
SVC				
weighted AraVec-CBoW	0.836	0.795	0.811	0.911
CNN				
AreVec-SG	0.825	0.845	0.834	0.938
CNN				
weighted AraVec-SG	0.851	0.819	0.832	0.926
CNN				
AreVec-CBoW	0.829	0.791	0.806	0.922
CNN				
weighted AreVec-CBoW	0.812	0.694	0.717	0.898
LSTM				
AreVec-SG	0.832	0.840	0.836	0.944
LSTM				
weighted AreVec-SG	0.865	0.842	0.853	0.940
LSTM				
AreVec-CBoW	0.837	0.831	0.834	0.927
LSTM				
weighted AreVec-CBoW	0.823	0.788	0.802	0.913

## Data Availability

The data presented in this study are available upon request from the author. The data are not publicly available as this research is ongoing.
